# Retrospective analysis of urinary tract stone composition in a Chinese ethnic minority colony based on Fourier transform infrared spectroscopy

**DOI:** 10.1038/s41598-023-40603-w

**Published:** 2023-08-18

**Authors:** Junfeng Zhang, Kailing Li, Hongbo Chen, Xiaohui Hu, Zicheng Guo, Su Chen, Fu Zheng, Wusong Cheng, Qian Mu, Yong Lan, Peng Chen

**Affiliations:** 1 Department of Urology, Affiliated Tumor Hospital of Xinjiang Medical University, Urumqi, China; 2 Department of Urology, The Central Hospital of Enshi Tujia and Miao Autonomous Prefecture, No. 158 Wuyang Avenue, Enshi City, 445000 Hubei China

**Keywords:** Diseases, Kidney diseases, Urology

## Abstract

To analyze the relationship between the composition of urinary stones and various influencing factors in the Enshi region. We used FT-IR to examine the composition of 1092 stone samples. Combined with the relevant clinical materials, the data were analyzed using both one-dimensional statistical methods and multivariate statistical methods. The study included 1092 stone samples, classified as follows: 457 (41.8%) with a single component, 453 (41.5%) with two components, 149 (13.6%) with three components, and 33 (3.0%) with four components. Stones were categorized into five types: Calcium Oxalate (CaOx) (76.4%), carbapatite (CaP) (9.3%), Struvite (ST) (8.3%), Uric Acid (UA) (4.9%), and Others (1.0%). Age, gender, urinary tract infection (UTI), family history of urinary stones (FH), hyperuricemia (HUA) and stone location were significantly associated with stone type. Logistic regression revealed that females and UTI were relative risk factors for predicting CaP and ST, while FH and HUA were relative risk factors for predicting UA. Our study indicates that the overall composition of urinary tract stones in the Enshi region is consistent with that of the entire China. Additionally, the predisposing factors for stone formation vary in terms of gender, age, FH, UTI, hyperuricemia HUA, and stone location.

## Introduction

In the past few decades, urolithiasis has become an increasingly common disease, with a rising global incidence, prevalence, and recurrence rates^1^. The causes of urolithiasis are complex and closely related to various factors, such as race, gender, age, genetics, environment, and diet^2^. Despite the common features of urolithiasis worldwide, unique variants exist in certain countries and regions. For instance, the incidence of urologic stones globally ranges from 1 to 5%^3^, while in the southern region of China, it is notably higher at 5–10%, with recurrence rates of 50% within five years and 75% within twenty years^4^. In 2019, the Urolithiasis Group of the Chinese Urological Association (CUA) conducted a prospective nationwide multicenter survey, investigating the impact of gender, age, body mass index (BMI), stone location, and geographical region on the diversity of urinary stone composition^5^. In Enshi Prefecture, Southwest China, urinary stone disease poses a significant health concern. Due to its status as a minority-inhabited area, the region's unique geographical environment, lifestyle habits, and climatic conditions may lead to distinct characteristics in the development of urinary stones. Accurate analysis of urinary stone composition is crucial for identifying stone etiology, prevention, and treatment^6^. However, there is currently no research conducted to determine the specific features of urinary stone composition in this region.

Fourier transform infrared spectroscopy (FT-IR) has emerged as a sensitive, reliable, and accurate method for detecting urinary stones, and it has become the standard diagnosis process^7,8^. FT-IR utilizes distinctive infrared absorption patterns exhibited by different chemical compounds to precisely identify and characterize stone components^7,9^. Its high sensitivity allows for the detection of trace elements, and its rapid analysis capabilities require minimal sample preparation^10^. By comparing the spectra of unknown stones to reference spectra, FT-IR enables accurate determination of stone composition and type^11^. The application of FT-IR in urinary stone analysis has significantly advanced our understanding of stone formation, composition, and recurrence patterns.

To better comprehend the composition of urinary tract stones and their association with various influencing factors in the Enshi region, we conducted a retrospective analysis of 1092 stone specimens using FT-IR. These stone specimens were collected from the Department of Urology at the Central Hospital of Enshi Tujia and Miao Autonomous Prefecture from January 2019 to August 2022. In this study, we explore whether differences in the stone composition may be explained by differences in gender, age, diabetes mellitus (DM), hypertension (HTN), urinary tract infections (UTI), family history of urinary stones (FH), hyperuricemia (HUA), and anatomical locations using univariate statistical analysis (The abbreviation table at the end of the document presents common abbreviations and their full forms). Additionally, we established an unordered multi-categorical logistic statistical model to discern the relative risk factors associated with less common stone types. Ultimately, this study seeks to offer more scientifically accurate urinary stone prevention strategies tailored to the specific population in the Enshi region.

## Patients and methods

### Stone composition analyses and classification

The study received approval from the Ethics Committee of the Central Hospital of Enshi Tujia and Miao Autonomous Prefecture (NO. 2022-013-01). Between January 2019 and August 2022, a total of 1092 urinary stone samples and associated clinical information were included in the study. The patients were long-term residents (≥ 5 years) in the Enshi area, experiencing urinary stones symptoms for the first time and undergoing surgical treatment. Stone fragments were collected intraoperatively during percutaneous nephrolithotomy (PCNL), ureteroscopic lithotripsy, and cystolithotripsy. Clinical and demographic data, including age, gender, stone anatomical localization, geographical region, and underlying disease, were obtained. For stone sample analysis, FT-IR (Dingshun, SUN-3G, Jinan, China) was employed, following standard operating procedures established by manufacturers and hospitals. Stone samples were split, washed, and dried in a 100 °C oven for 2–3 min. The stone powder (1.5 mg) was then mixed with potassium bromide (200 mg) and ground to the micrometer level using an agate mortar and pestle. The mixed powder was pressed into a uniform and translucent sheet using a tablet press mold and tablet press (16 Mpa, stop 5 s) as per the manufacturer's instructions. In addition, a potassium bromide tablet (without stones) was prepared as a control. The assay parameters were set at a sampling resolution of 2 cm^−1^, a spectral range of 4000–400 cm^−1^, and a total of 32 scans. Before sample analysis, the instrument background was measured using potassium bromide tablets (without stones). Subsequently, the stone samples were inserted into the sample compartment, and the FT-IR instrument recorded the transmittance spectrum, generated the infrared spectrum, automatically analyzed the composition of the stones, and generated a corresponding test report.

The stone composition was classified following to the European Association of Urology stone classification practices^6^. The most common form of stone is calcium oxalate (CaOx), with two types: monohydrate (COM) and dihydrate (COD)^12^. Stones containing over 50% calcium oxalate were classified as CaOx stones. Uric acid stones (UA) included stones with more than 50% uric acid and uric acid dihydrate. Stones with over 50% carbapatite were classified as carbapatite (CaP) stones, while those with more than 50% struvite were labeled as struvite uroliths (ST). Cystine stones encompassed stones containing cystine. For statistical analysis, rare stones such as cystine stones and drug-related stones were grouped into the "Other" category.

### Ethics statement

The experiments and procedures were conducted following the relevant guidelines and regulations. Ethical approval was obtained from The Central Hospital of Enshi Tujia and Miao Autonomous Prefecture (Approval No. 2022-013-01). All patients provided informed consent to participate in the study.

### Consent for publication

Informed consent was obtained from all participants involved in the study. For participants under the age of 16 years, informed consent was obtained from their parents and/or legal guardians.

### Statistical analyses

Quantitative data were presented as mean ± standard deviation. Qualitative data were expressed as n (%). The chi-squared test was employed to evaluate the impact of gender, DM, HTN, and UTI on different types of urinary stones. In cases where the chi-squared test was not applicable, Fisher's exact test was utilized for variables FH and HUA. Cramer's V coefficient was employed to assess the strength of correlation between categorical variables, with values between 0.1 and 0.3 indicating weak correlation, between 0.3 and 0.5 indicating moderate correlation, and greater than 0.5 indicating strong correlation. Adjusted standardized residuals (Adj.R) were used to assess the association between two categorical variables, with values greater than 3 indicating significant deviations between observed and expected values, signifying a notable correlation between the two variables. The Kruskal–Wallis’s test was conducted for unidirectional ordered data (age groups), followed by Z-tests for multiple comparisons, with p-values adjusted using Bonferroni correction. Unordered multi-categorical logistic regression was performed to evaluate the influence of gender, age, DM, HTN, UTI, FH, HUA, and stone anatomical location on stone composition. Statistical analyses were conducted using SPSS software (version 23, IBM Corp., Armonk, NY, USA), and a p-value (two-tailed) < 0.05 was considered statistically significant. Data visualization and graph plotting were performed using ORIGINE software (version 2022 Pro, OriginLab Corporation, Northampton, MA, USA).

## Results

### Individual and clinical characteristics of the patients

**Figure 1:**
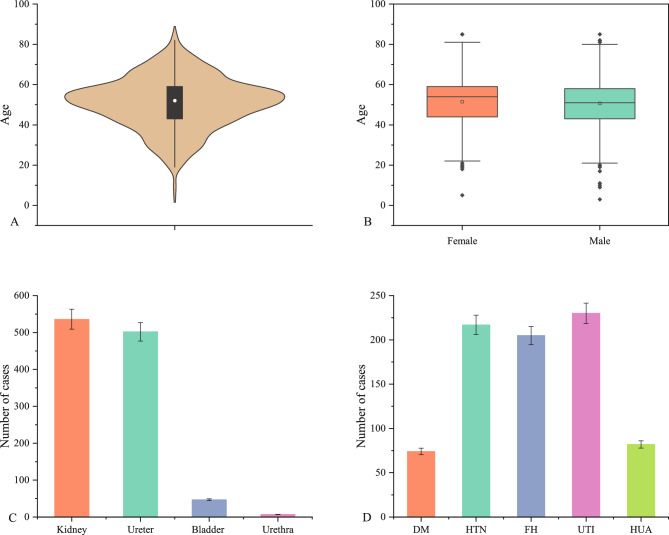
The basic information and general clinical of the patients. (**A**) The study included a total of 1092 patients, ranging in age from 3 to 85 years (mean age: 50.9 ± 12.8 years, median age: 52 years). (**B**) Among the patients, 745 (68.2%) were males and 347 (31.8%) were females, with no significant difference in age between males (mean age: 50.6 ± 12.9 years) and females (mean age: 51.4 ± 12.8 years) (t = -1.027, v = 1090, p > 0.05). (**C**) The distribution of stone anatomical locations was as follows: kidney—536 (49.5%), ureter—502 (46.0%), bladder—47 (4.3%), and urethra—7 (0.6%). (**D**) Distribution of underlying diseases: DM (diabetes mellitus)—74 (6.8%), HTN (hypertension)—217 (19.9%), FH (family history of urinary stones)—205 (19.8%), UTI (urinary tract infection)—230 (21.0%), and HUA (hyperuricemia)—82 (7.5%).

A total of 1092 patients were included in the study, with 745 (68.2%) men and 347 (31.8%) women, resulting in a male-to-female ratio of 2.1:1. The age of the patients ranged from 3 to 85 years, with a mean age of 50.9 ± 12.8 years. There were no significant differences in age between males (50.6 ± 12.9 years) and females (51.4 ± 12.8 years) (t = − 1.027, v = 1090, p > 0.05). The distribution of urinary stone locations among the patients was as follows: 536 (49.1%) cases of renal calculi, 502 (46.0%) cases of ureteral calculi, 47 (4.3%) cases of bladder calculi, and 7 (0.6%) cases of urethral calculi. Moreover, 74 (6.8%) patients had diabetes mellitus (DM), 217 (19.9%) patients had hypertension (HTN), 205 (19.8%) patients had a family history (FH) of urinary stones, 230 (21.0%) patients had urinary infections (UTI), and 82 (7.5%) patients had hyperuricemia (HUA) (Fig. 1).

### General information about the stone composition

**Figure 2:**
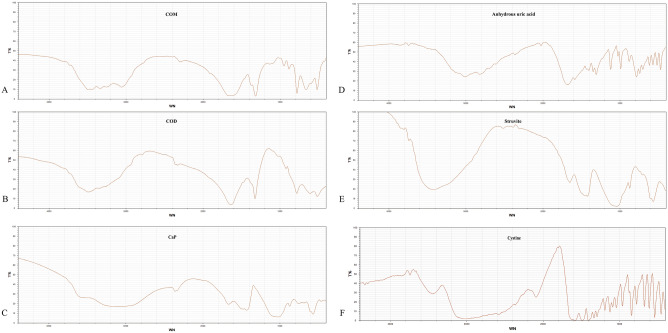
Characteristic infrared spectra of different stone components. The red lines represent downward absorption peaks (negative peaks). The x-axis indicates the wavelength (cm^−1^), representing the position of absorption peaks, while the y-axis shows transmittance (T%), indicating the absorption intensity. (**A**) Calcium oxalate monohydrate (COM) exhibits absorption peaks at 3480–3050 cm^−1^, 1620 cm^−1^, 1320 cm^−1^, 950 cm^−1^, 885 cm^−1^, 780 cm^−1^, and 655 cm^−1^ in the infrared spectrum. The peaks at 1620 cm^−1^ and 1320 cm^−1^ are attributed to the C = O and O–H vibrations, respectively. The broad absorption peak at 3480–3050 cm^−1^ corresponds to water molecule vibrations, which are split into five individual peaks. (**B**) In comparison, calcium oxalate dihydrate (COD) exhibits a broad absorption peak at 3460 cm^−1^ in the infrared spectrum, without any splitting phenomenon. Additionally, there is no absorption peak at 665 cm^−1^. (**C**) The infrared spectrum of calcium phosphate (CaP) shows absorption peaks at 1630, 1460, 1415, 1100–1000, 870, 605, 570, and 450 cm^−1^. (**D**) Anhydrous uric acid's infrared spectrum displays absorption peaks at 1650, 1590, 1400, 1345, 1300, 1120, 1025, 900, 785, 700, 620, and 575 cm^−1^. (**E**) The infrared spectrum of struvite exhibits absorption peaks at 3500–3000, 1650, 1440, 1400, 1100–1000, 750, and 570 cm^−1^. (**F**) Cystine stones exhibit absorption peaks mainly at 1600–1200, 1125, 1020, 965, 850, 780, 680, 610, and 530 cm^−1^ in the infrared spectrum.

**Figure 3:**
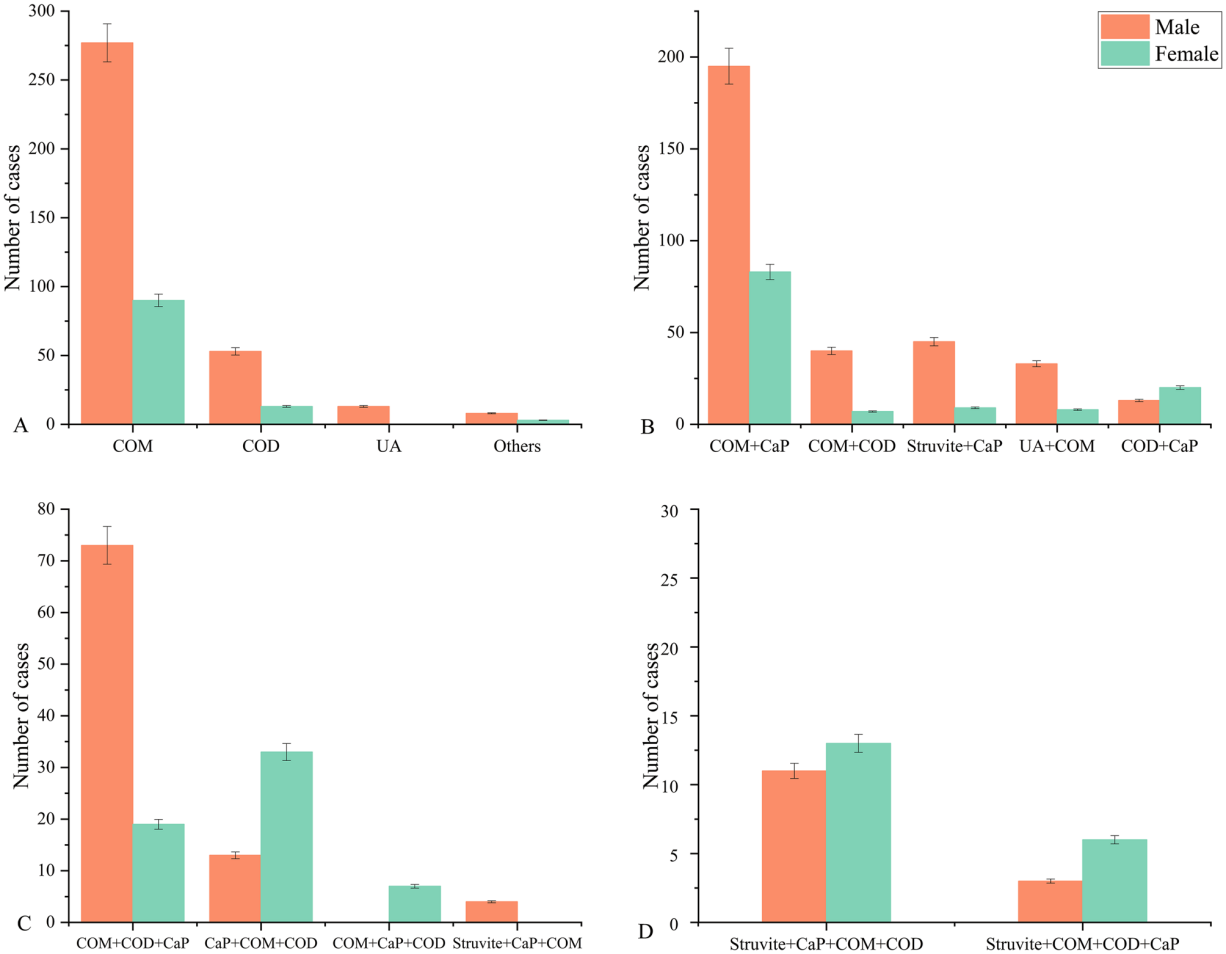
General overview of the composition of urinary stones. The error bars represent standard deviation. From A to D, respectively, it represents the number and proportion of single-component stones, two-component stones, three-component stones, and four-component stones in the total, as well as the number and proportion of males and females.

Infrared spectra of substances are unique, akin to human fingerprints^13–15^. Consequently, distinct components of urinary tract stones exhibit characteristic infrared spectra, indicating their specific compositions. By utilizing these characteristic spectra (Fig. 2), we conducted qualitative and quantitative analyses of different stone compositions. A total of 1092 stone samples underwent FT-IR analysis for stone composition. Quantitative analysis revealed that single-component samples constituted 457 (41.8%) cases, including 367 (33.6%) cases of COM, 66 (6.0%) cases of COD, 13(1.2%) cases of UA, and 11(1.0%) cases of Others (rare components: L-cystine, drug-induced stones). Stones composed of two components accounted for 453 (41.5%) cases, comprising 278 (25.4%) cases of COM + CaP, 47 (4.3%) cases of COM + COD, 54 (4.9%) cases of Struvite + CaP, 41(3.8%) cases of UA + COM, and 33(3.0%) cases of COD + CaP. Stones with three components were observed in 149 (13.6%) cases, including 92 (8.4%) cases of COM + COD + CaP, 46 (4.2%) cases of CaP + COM + COD, 7(0.6%) cases of CaP + COM + COD, and 4 (0.4%) cases of Struvite + CaP + COM. Additionally, stones with four components were found in 33(3.0%) cases, comprising 24 (2.2%) cases of Struvite + CaP + COM + COD and 9 (0.8%) cases of Struvite + COM + COD + CaP (Fig. 3).

**Figure 4:**
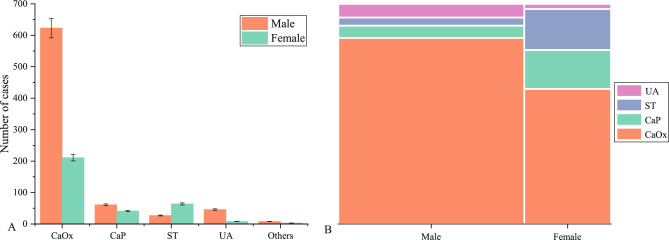
Distribution of different urinary stone types. (**A**) The distribution of five stone types, with 843 cases of CaOx, 102 (9.3%) cases of CaP, 91(8.3%) cases of ST, 54 (4.9%) cases of UA, and 11(1.0%) other rare stone types. The error bars represent standard deviation. (**B**) The chi-square test for differences in the distribution of gender and the four main types of stones among the 1081 study subjects (χ^2^ = 122.5, *p* < 0.001; Cramer's V = 0.337,* p* < 0.05).

Based on the stone classification principle described above, we categorized all stones into five types: CaOx, CaP, ST, UA, and Others (Fig. 4A). CaOx was the most prevalent stone type, with a total of 834(76.4%) cases, including 623(57.1%) in males and 211 (19.3%) in females. It was followed by 102 (9.3%) cases of CaP, including 61(5.6%) cases in males and 41(3.7%) cases in females. A total of 91(8.3%) cases of ST were detected, comprising 27 (5.8%) cases in males and 64(2.5%) cases in females. Among the 54 (4.9%) cases of UA, 46 (4.2%) were male and 8 (0.7%) were female. Additionally, there were 11(1.0%) other rare stone types, including 8(0.07%) in males and 3(0.03%) in females, notably 3 of which were drug-induced stones in children.

### The relationship between gender and four types of urinary stones

**Figure 5:**
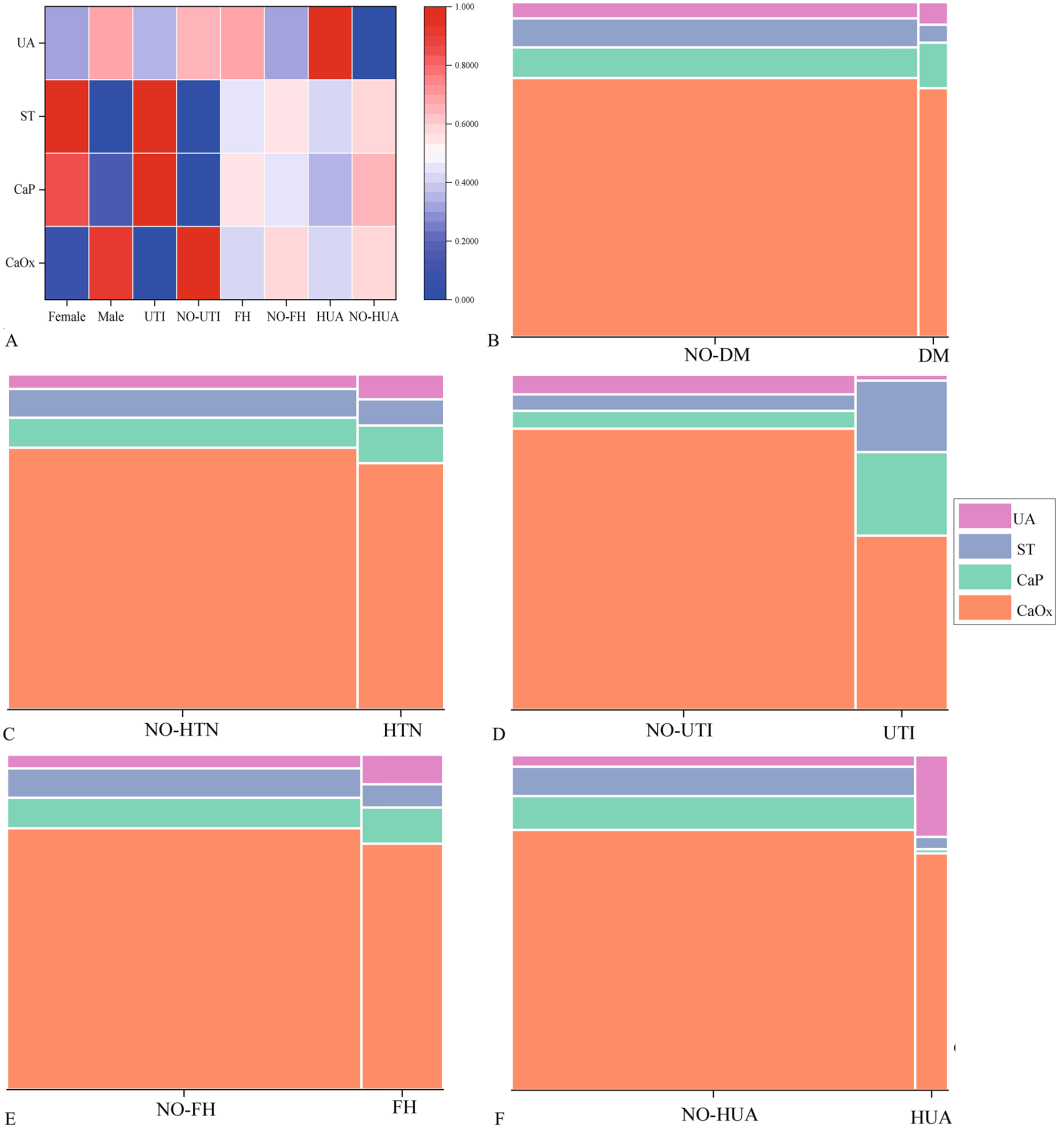
The analysis of the relationship between different factors and the four main types of urinary stones (CaOx, CaP, ST, UA). (**A**) The heat map displays the normalized Adj.R, with higher convergence to red indicating a stronger association. The red color highlights that women are more likely to develop ST and CaP stones, while men are more prone to CaOx stones. Additionally, patients with UTI have a higher tendency to develop ST stones, and those with HUA are more likely to have UA stones. (**B–F**) Mosaic plots were used to display the results of Chi-square tests or Fisher's exact tests, which were conducted to examine the differences between underlying diseases and urinary stone types, two categorical variables. Each color block represents a specific type of urinary stone, and the size of the block corresponds to the number of stones in that category. The specific test results are as follows: (**B**) DM vs NO-DM: χ^2^ = 2.8, *p* = 0.418; Cramer's V = 0.051, *P* > 0.05; (**C**) HTN vs NO-HTN: χ^2^ = 4.6, *P* = 0.202; Cramer's V = 0.065, *P* > 0.05; (**D**) UTI vs NO-UTI: χ^2^ = 161.7, *p* < 0.001; Cramer's V = 0.387,* p* < 0.001; (**E**) FH vs NO-FH: p = 0.037 < 0.05; Cramer's V = 0.092,p > 0.05; (**F**) HUA vs NO-HUA:* p* < 0.001; Cramer's V = 0.265, p < 0.001).

Because the “Other” stone types are rare and inclusion in statistical models can produce extreme values that affect the accuracy of statistical results, we do not include this type in some statistical models for analysis. A total of 1081 cases were included in the statistical model, which analyzed the relationship between sex and four main types of stones (CaOx, CaP, ST, UA). The results show that any of the expected frequencies are greater than 5, and the chi-square test can be used, χ^2^ = 122.5, *p* < 0.001, suggesting that the four main types of stones differ significantly from gender type. There is a moderately strong correlation between four different stone types and gender (Cramer's V = 0.337, *p* < 0.05) (Fig. 4B). We conducted a more in-depth analysis of the above results using post hoc testing and assessed the relationship between the two categorical variables based on the adjusted standardized residuals (Adj.R). In the CaOx group, 623 (57.6%) were males and the Adj. R value of 8.5 indicated a significant association between CaOx stones and male gender, suggesting a higher likelihood of CaOx stones in male patients compared to females. Similarly, in the CaP group, there were 61 (5.6%) females, and the Adj.R value of 6.4 revealed a significant association between CaP stones and the female gender. Likewise, in the ST group, there were 64 (5.9%) females, and the Adj.R value of 8.2 also indicated a significant association between ST stones and female gender. These results consistently suggest a higher likelihood of CaP and ST stones in female patients compared to males (Fig. 5A).

### The relationship between DM, HTN, FH, UTI, HUA and urinary stone types

A total of 1081 cases were included in the statistical model, which examined the difference between DM, HTN, FH, UTI, HUA, and four main types of stones (CaOx, CaP, ST, UA). The results showed that for DM, HTN, and UTI, all expected frequencies were greater than 5, allowing the use of the chi-square test. From the perspective of DM (χ^2^ = 2.8, *p* = 0.418; Cramer's V = 0.051, *p* > 0.05) and HTN (χ^2^ = 4.6, *p* = 0.202; Cramer's V = 0.065,* p* > 0.05), there was no statistically significant difference, indicating that the presence or absence of DM/HTN did not significantly affect the type of stone (Fig. 5B,C). However, among the 1081 study participants, 230 had UTI, and the data (χ^2^ = 161.7, *p* < 0.001) indicated a significant difference in the distribution of the four different stone types based on the presence or absence of UTI. There was a moderately strong correlation between four different stone types and UTI, with Cramer's V = 0.387, *P* < 0.001 (Fig. 5D). Further post hoc testing revealed that patients with UTI were more likely to develop CaP (Adj.R = 9.0) and ST (Adj.R = 8.0) stones (Fig. 5A).

Among the 1081 study participants, 205 had FH, and 82 had HUA. Due to expected frequencies of less than 5, Fisher's exact test was chosen. The results indicated a significant difference between FH and the four different stone types (*p* < 0.05), but there was no strong correlation between them (Cramer's V = 0.092, *p* > 0.05) (Fig. 5E). Furthermore, the test results showed a clear difference between HUA and different types of stones (*p* < 0.001), and there was a weak correlation between four different stone types and HUA, with Cramer's V = 0.26, *p* < 0.001 (Fig. 5F). Post hoc testing revealed that patients with Hyperuricemia were more likely to develop UA stones (Adj.R = 8.4) (Fig. 5A).

### The relationship between age and urinary stone types

**Figure 6:**
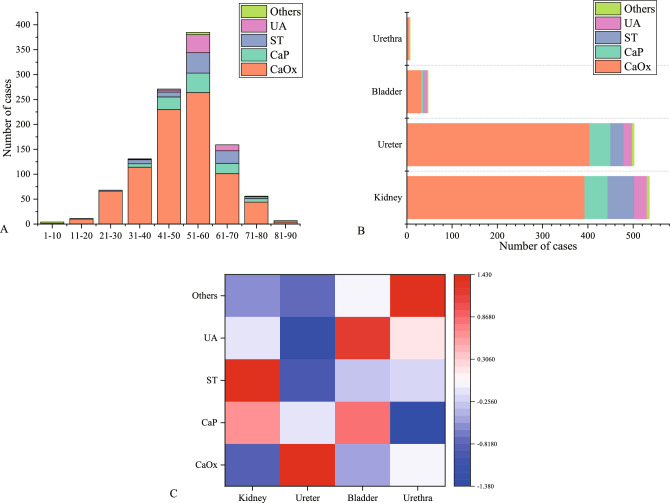
Distribution of different stone types across age groups and stone locations. (**A**) The distribution of different stone types across age groups. Kruskal–Wallis tests (H = 78.388, p < 0.001) revealed significant differences between different age groups and stone types. (**B**) Significant differences in the distribution of different stone types among different anatomical sites (Fisher's exact test, p < 0.001; Cramer's V = 0.108, p < 0.001). (**C**) The normalized Adj.R values were represented on a heat map, where a higher convergence to red indicates a stronger association. The red color indicates that ST is more likely to be found in the kidney, CaOx in the ureter, UA in the bladder, and rare stones in the urethra, relative to other stone types.

The relationship between age and urinary stone types was examined by dividing all study participants into 9 age strata, each representing a 10-year age group. This division allowed for a comprehensive analysis of the association between age and different stone compositions. Using Kruskal–Wallis tests, we found a statistically significant difference between the various age groups and stone types (H = 78.388, p < 0.001) (Fig. 6A). Further multiple comparisons were performed using the Z test with Bonferroni-adjusted p-values, revealing specific patterns. The proportion of CaOx cases was higher in the age groups of 21–30 years (compared to age groups 1–10, 51–60, 61–70, 71–80, 81–90 years, p < 0.05), 31–40 years (compared to age groups 1–10, 51–60, 61–70 years, p < 0.05), and 41–50 years (compared to age groups 1–10, 51–60, 61–70 years, p < 0.05) compared to other types of stones. This suggests that individuals in these age groups are more likely to develop CaOx stones.

In the 51–60 and 61–70 age groups, the proportion of ST cases (compared to age group 41–50 years, p < 0.05; 61–70 years compared to age group 41–50 years, p < 0.05) and UA cases (compared to age groups 31–40, 41–50 years, p < 0.05; 61–70 years compared to age group 41–50 years) was higher than other stone types. This indicates a higher likelihood of developing ST and UA stones in these age groups. Furthermore, we observed that rare stones were more likely to affect children aged 1–10 (compared to age groups 31–40, 41–50, 51–60, p < 0.05). Additionally, individuals aged 81–90 (compared to age group 31–40, p < 0.05) were most likely to develop CaP stones (Table 1).In summary, age appears to play a significant role in the distribution of different stone types, with distinct patterns observed in various age groups.

**Table 1:** Crosstabulation of stone type and age.

Stone type	Age								
	1–10	11–20	21–30	31–40	41–50	51–60	61–70	71–80	81–90
CaOx	1_a_	10_a,b_	66_b_	114_b,c,e,f_	230_b,d,e,f_	264_a_	101_a_	44_a,e_	4_a,f_
CaP	0	0	0	7_a_	25_a,b_	39_a,b_	21_a,b_	7_a,b_	3_b_
ST	0	1_a,b_	2_a,b_	8_a,b_	10_a_	41_b_	25_b,c_	4_a,b_	0
UA	0	0	0	1_a,c_	4_a_	36_b_	12_b,c_	1_a,b_	0
Others	3_a_	0	0	1_b_	2_b_	5_b_	0	0	0

*Note* The number of cases is denoted by n, in the two-sided equivalence test for column proportions, values that do not share the same subscripts in the same row and sub-table are significantly different at p < 0.05.

### The relationship between stone location and urinary stone types

In the analysis of 1092 stone samples, due to the expected frequencies being less than 5, we opted for Fisher's exact test. The results revealed a significant difference (*p* < 0.001) in the distribution of stone types based on their locations. Furthermore, there was a weak correlation (Cramer's V = 0.108, *p* < 0.001) between different stone types and their locations (Fig. 6B). Subsequent post hoc testing indicated that ST tended to occur more frequently in the kidneys (Adj.R = 3.1), UA tended to occur more in the bladder (Adj.R = 3.2), and rare stones tended to occur in the urethra (Adj.R = 3.5) (Fig. 6C).

### Relative risk analysis for non-CaOx Stone types using unordered multi-classification logistic regression

In this study, we employed an unordered multi-classification Logistic regression model with four different stone types (CaOx, CaP, ST, UA) as the unordered multi-class response variables. Simultaneously, we considered six statistically significant factors (gender, age, family history, urinary tract infection, hyperuricemia, and stone location) from the previous research as independent variables. As CaOx had the highest number of cases, it served as the reference category. The Logistic regression model was utilized to predict and assess the relative risk factors for stone types other than CaOx in specific populations. Compared to CaOx, we observed that female gender (RR= 3.087, 95% CI 1.944–4.903) and urinary tract infection (RR= 5.272, 95% CI 3.219–8.635) were relative risk factors for predicting CaP. Similarly, female gender (RR= 4.871, 95% CI 2.944–8.060) and urinary tract infection (RR= 4.921, 95% CI 2.910–8.323) were relative risk factors for predicting ST. Additionally, family history (RR= 2.539, 95% CI 1.286–5.012) and hyperuricemia (RR= 7.729, 95% CI 4.010–14.898) were relative risk factors for predicting UA (Tables 2, 3).This model enables us to understand the relative risk factors associated with specific factors and different stone types, thus providing better support for predicting and implementing personalized treatment approaches for individuals with specific urinary stone types.

**Table 2:** Variable assignment for unordered multi-classification logistic regression.

Factors	Variables	Variable assignment
Type of stone	Y	CaOx = 0, CaP = 1, IS = 2, UA = 3
Gender	X1	Female = 0, Male = 1
Age	X2	1–30 = 1,31–60 = 2,61–90 = 3
FH	X3	NO- FH = 1, FH = 0
UTI	X4	NO- UTI = 1, UTI = 0
HUA	X5	NO- HUA = 1, HUA = 0
Stone location	X6	Urethra = 1, Bladder = 2, Ureter = 3, Kidney = 4

**Table 3:** Unordered multi-classification logistic regression.

*Model*		β	SE	Wald	P vaule	Exp(β)	95%CI
Logit P_CaP/CaOx_	Intercept	− 2.600	1.110	5.487	0.019		
	Female	1.127	0.236	22.805	0.000	3.087	1.944 ~ 4.903
	UTI	1.662	0.252	43.620	0.000	5.272	3.219 ~ 8.635
Logit P_ST/CaOx_	Intercept	− 1.965	0.787	6.241	0.012		
	Ureter	− 0.868	0.260	11.128	0.001	0.420	0.252 ~ 0.699
	Female	1.583	0.257	37.985	0.000	4.871	2.944 ~ 8.060
	UTI	1.594	0.268	35.331	0.000	4.921	2.910 ~ 8.323
Logit P_UA/CaOx_	Intercept	− 2.457	0.386	40.432	0.000		
	HUA	2.045	0.335	37.297	0.000	7.729	4.010 ~ 14.898
	FH	0.932	0.347	7.211	0.007	2.539	1.286 ~ 5.012

The reference category is CaOx.

## Discussion

In this study, we aimed to investigate the relationship between disease-related factors and urinary stone composition or types in the Enshi region. Urinary stones' composition and types vary in different countries and regions due to multiple etiological factors^16,17^. Currently, FT-IR is the most widely used method for detecting urinary stone components^18,19^. Therefore, FT-IR was employed to analyze 1092 urinary stone samples, revealing 457 single-component stones and the remainder being mixed stones (Fig. 3). The most frequent component was CaOx, accounting for 76.4% of cases, followed by CaP (9.3%), ST (8.3%), UA (4.9%), and Others (1.0%) (Fig. 4A). The study included 745 males and 347 females, with an approximate male to female ratio of 2.1:1, which is similar to previous studies in other regions of Korea^20^, India^21^, the United Kingdom^16^, and Brazil^17^. Our results showed statistically significant differences (*p* < 0.001) and moderate correlations (Cramer's V = 0.337, *p* < 0.05) between gender and the four main stone types. CaOx stones are more prevalent in men, whereas ST and CaP are more common in women (Figs. 4B and 5A). Similar gender differences in stone types were observed in a multicenter study across China^5^. The male predisposition to CaOx may be attributed to the following factors: the influence of testosterone and dihydrotestosterone on CaOx crystal formation in rat models^22^, the promotion of CaOx crystal formation by enhanced androgen receptor (AR) signaling in hepatocytes^23,24^, as well as factors such as heavy physical work, excessive sweating, inadequate fluid intake, and concentrated urine volume^25^. Furthermore, the incidence of UA was higher in men compared to women, with a male-to-female ratio of 5.75:1, which is consistent with previous studies^26,27^. Additionally, our study aligns with previous research, indicating a higher incidence of ST and CaP in women^17^.

The highest prevalence of urinary stones was observed in the age group of 13–60 years, with a distinct peak distribution (Fig. 6A). Our analysis revealed significant differences in the distribution of five stone types across nine age groups (p < 0.001). Individuals aged 31–60, who constitute the main productive force in society, showed higher susceptibility to CaOx stones, likely due to factors such as heavy physical labor leading to increased sweating and inadequate water intake, particularly in economically disadvantaged rural areas^5,28^. Additionally, the risk of UTI tends to increase in women during menopause after the age of 50, when estrogen levels significantly decline, which may contribute to the development of infectious stones in older women^29^. Our study observed similar trends to previous research conducted in China^5,18^ and other countries^26,30^, we further employed the Kruskal–Wallis test and multiple comparison tests. This approach allowed us to offer a more detailed and comprehensive statistical analysis of the variations in stone occurrence among different age groups (Fig. 6A and Table 1).

Indeed, urolithiasis has been associated with metabolic syndrome, suggesting it may result from interactions between various metabolic risk factors^31^. We observed that patients with DM and HTN did not exhibit a specific predisposition to any particular type of stone (*p* > 0.05), while HUA was significantly associated with an increased risk of UA formation (*p* < 0.05) (Fig. 5). UA stones are strongly associated with causative factors such as dietary habits (red meat, seafood, beer), hyperuricemia, urine pH, hypercholesterolemia, obesity and insulin resistance^34,35^. Studies in China have demonstrated that patients with metabolic syndrome have a significantly increased risk of developing urolithiasis^32,33^. The complex interplay between metabolic factors and stone formation requires further exploration to better understand the underlying mechanisms. Furthermore, our study showed significant variability (p < 0.001) and correlation (Cramer's V = 0.108) between stone locations and types. ST stones were more likely to occur in the kidney (Adj. R = 3.1), UA stones in the bladder (Adj. R = 3.2), and rare stones in the urethra (Adj. R = 3.5) compared to the other anatomical locations (Fig. 6B,C). Consistent with previous studies in China^5,18,34^, the most common stones in the kidney (36.0%) and ureter (36.9%) from a composition ratio standpoint were CaOx stones. The relationship between stone location and stone composition can reflect part of the causes of stone formation: bladder stones are significantly associated with urinary tract obstruction, nutritional deficiencies, and uric acid excretion, and therefore uric acid stones tend to form there^35^.

It is widely known that CaOx stones constitute the largest proportion of patients and that patients have the highest probability of getting CaOx stones^30,36^. However, predicting the type of stone with a lower composition ratio or probability of occurrence in patients with urinary stones has greater challenges and implications. We performed a multifactorial logistic regression analysis with CaOx as the reference group, identifying female gender and urinary tract infection as relative risk factors for CaP and ST, and FH and HUA as relative risk factors for UA stones (Tables 2, 3).

Our study has some limitations. First, we only focused on the disease characteristics in the local area of Enshi, which is not widely representative. Second, our data did not study the urine biochemical characteristics and some serum characteristics of patients. Third, information bias inevitably causes some errors, such as possible recall of information provided by patients when collecting relevant disease information. We provide valuable insights into the characteristics of urinary stones in the Enshi region. The consistency of our findings with studies from other regions in China highlights the universality of certain patterns of urinary stone prevalence nationwide. However, it is important to recognize that regional differences and local risk factors may still influence the patterns of stone formation. Regional variations and local risk factors require longer-term and larger cohort observations for further investigation.

In summary, our study comprehensively investigated the relationships between individual characteristics, disease history, stone location, and stone composition. Based on our findings, targeted management and prevention strategies can be developed for specific populations in the region. In future research, exploring stone composition differences among different regions and populations to understand regional and individual influences on stone formation is crucial. Additionally, delving into the association between metabolic factors and stone formation, considering lifestyle, dietary habits, and genetic factors, is important. Improved study designs and methods, such as larger cohort observations, multicenter studies, and molecular biology techniques, will further enhance stone research and provide more effective prevention and treatment strategies for patients.

## Data Availability

On reasonable request, the corresponding author will provide the datasets used and analyzed in this study.

## Abbreviations

FT-IR: Fourier transform infrared spectroscopy
CaOx: Calcium oxalate stone
COM: Calcium oxalate monohydrate
COD: Calcium oxalate dihydrate
CaP: Carbapatite
ST: Struvite
UA: Uric acid stones
Other: Rare stones such as cystine
DM: Diabetes mellitus
HTN: Hypertension
UTI: Urinary tract infection
HUA: Hyperuricemia
FH: Family history of urinary stones
Adj.R: Adjusted standardized residuals
